# Anti-Arrhythmic Effect of Verapamil Is Accompanied by Preservation of Cx43 Protein in Rat Heart

**DOI:** 10.1371/journal.pone.0071567

**Published:** 2013-08-12

**Authors:** Peng Zhou, Shu-Miao Zhang, Qiu-Lin Wang, Qi Wu, Mai Chen, Jian-Ming Pei

**Affiliations:** 1 Department of Cardiology, Chengdu Medical College, Chengdu, P.R. China; 2 Department of Physiology, Fourth Military Medical University, Xi’an, P.R. China; 3 Department of Cardiology, Xijing Hospital, Fourth Military Medical University, Xi’an, P.R. China; Universidade Federal do Rio de Janeiro, Brazil

## Abstract

The present study was to test the hypothesis that anti-arrhythmic properties of verapamil may be accompanied by preserving connexin43 (Cx43) protein via calcium influx inhibition. In an *in vivo* study, myocardial ischemic arrhythmia was induced by occlusion of the left anterior descending (LAD) coronary artery for 45 min in Sprague-Dawley rats. Verapamil, a calcium channel antagonist, was injected i.v. into a femoral vein prior to ischemia. Effects of verapamil on arrhythmias induced by Bay K8644 (a calcium channel agonist) were also determined. In an *ex vivo* study, the isolated heart underwent an initial 10 min of baseline normal perfusion and was subjected to high calcium perfusion in the absence or presence of verapamil. Cardiac arrhythmia was measured by electrocardiogram (ECG) and Cx43 protein was determined by immunohistochemistry and western blotting. Administration of verapamil prior to myocardial ischemia significantly reduced the incidence of ventricular arrhythmias and total arrhythmia scores, with the reductions in heat rate, mean arterial pressure and left ventricular systolic pressure. Verapamil also inhibited arrhythmias induced by Bay K8644 and high calcium perfusion. Effect of verapamil on ischemic arrhythmia scores was abolished by heptanol, a Cx43 protein uncoupler and Gap 26, a Cx43 channels inhibitor. Immunohistochemistry data showed that ischemia-induced redistribution and reduced immunostaining of Cx43 were prevented by verapamil. In addition, diminished expression of Cx43 protein determined by western blotting was observed following myocardial ischemia *in vivo* or following high calcium perfusion *ex vivo* and was preserved after verapamil administration. Our data suggest that verapamil may confer an anti-arrhythmic effect via calcium influx inhibition, inhibition of oxygen consumption and accompanied by preservation of Cx43 protein.

## Introduction

Ischemic heart disease-induced ventricular tachyarrhythmia is the main cause of sudden cardiac death [1], [2]. Great strides, in particular in regard to pharmacological therapy, have been made in the treatment of cardiac arrhythmia. Anti-arrhythmic drugs modulate or mediate sodium channel blockade, β-adrenergic blockade, potassium channel blockade and/or calcium channel blockade, according to the Vaughan-Williams classification system [3].

It is well documented that myocardial ischemia may lead to intracellular calcium overload, thus resulting in cardiac arrhythmias. Therefore, calcium channel blocking by calcium channel antagonists such as verapamil may prevent cardiac arrhythmias. It has been demonstrated that calcium overload may induce uncoupling of connexin43 (Cx43), which contributes to the occurrence of cardiac arrhythmia [4]–[6]. However, no reports in the literature have documented the effect of calcium channel blockage by verapamil on Cx43 protein; therefore, study on Cx43, a new therapeutic target against cardiac arrhythmias, may provide new insight for the understanding of calcium channel blockage. Gap junction remodeling, which is mainly formed by Cx43 in the ventricle, has been reported to contribute to the enhanced propensity toward arrhythmogenesis [4]. Previous data indicate that cardiac-specific knockout of Cx43 can lead to spontaneous ventricular arrhythmias with subsequent sudden cardiac death [5]. Cx43-deficient mice have been demonstrated to exhibit accelerated onset and enhanced incidence of ventricular arrhythmias induced by ischemia [6]. Furthermore, it has been demonstrated that efferent vagal nerve stimulation protects the heart against ischemia-induced arrhythmias accompanied by prevention of the loss of phosphorylated Cx43 [7]. Therefore, Cx43 may serve as a promising target in the treatment of cardiac arrhythmias.

Our previous work demonstrated that U50,488H, a selective κ-opioid receptor agonist, prevents against ischemia-induced arrhythmia [8], [9]. Recent work from our laboratory has documented that U50,488H can prevent incidence of arrhythmias via stabilization of Cx43 protein [10]. U50,488H also elicits the effect of calcium channel blockage like verapamil [11]; however, the effect of calcium channel blockage by verapamil on Cx43 is still unclear. Therefore, the present study was designed to determine the role of Cx43 protein in verapamil-mediated antiarrhythmic effects.

## Materials and Methods

### Materials

Verapamil and Bay K8644 were obtained from Tocris Cookson, Inc. (Ellisville, MO, USA). Heptanol was purchased from Sigma-Aldrich Corp. (St. Louis, MO, USA). ^43^Gap26 (H-Val-Cys-Tyr-Asp-Lys-Ser-Phe-Pro-Ile-Ser-His-Val-Arg-OH) were purchased from Biomatik Corporation (Wilmington, DE). Sources of antibodies were as follows: Cx43 mouse monoclonal antibody (Millipore, Billerica, MA, USA), tubulin mouse monoclonal antibody (Beyotime, Shanghai, China) and horseradish peroxidase-conjugated secondary antibodies (goat anti mouse; ZSGB-BIO Company, China).

### Animal Preparation

Adult male Sprague-Dawley (SD) rats (250−350 g) were used in the present study and were subjected to humane care according to institutional guidelines. This study conforms to the Guide for the Care and Use of Laboratory Animals published by the U.S. National Institutes of Health, NIH Publication No. 85–23, revised 1996. All of the animals were subjected to humane care according to protocols approved by Fourth Military Medical University Committee on Animal Care and Use. The Fourth Military Medical University Committee has specifically approved our study.

### *In vivo* Arrhythmia Study

SD rats weighing 250 to 350 g were anesthetized by i.p. injection of 3% pentobarbital (60 mg/kg). Supplemental doses of sodium pentobarbital were given as necessary to maintain a uniform level of anesthesia. Arrhythmia was induced by temporary occlusion of the left anterior descending (LAD) coronary artery from the area immediately below the left atrial appendage to the right portion of the left ventricle with a 6–0 silk suture for 45 min as described previously [10]. Mean arterial blood pressure (MABP) was continuously monitored via a saline-filled catheter (PE50, Becton Dickinson, Franklin Lakes, NJ) inserted into the right femoral artery, which was connected to a pressure transducer (AB-621G, Nihon Kohden, Tokyo, Japan). PE50 catheters were inserted into the left ventricle (LV) from the right carotid artery for the measurement of LV pressure (LVSP) with a pressure transducer (AB-621G, Nihon Kohden). Before and during ischemia, electrocardiogram (ECG) was used to measure heart rate (HR) and the incidence of arrhythmias including percent of animals with premature ventricular contractions (PVC), percent of animals with ventricular tachycardia (VT), and percent of animals with ventricular fibrillation (VF).

Verapamil (1 mg/kg [12]) was injected i.v. into a femoral vein 10 min prior to ischemia. A sham group underwent the same surgical procedures, except the suture underneath the LAD was left untied.

In another series of experiment, arrhythmia was induced by Bay K8644, an L-type calcium channel agonist, at a dose of 0.1 mg/kg given i.v. into the FV. Verapamil (1 mg/kg) was administered 10 min prior to Bay K8644. All injections were performed within 30 sec.

### Heart Isolation and Perfusion

SD rats weighing 250–350 g were anesthetized by i.p. injection of pentobarbital (40 mg/kg). Heparin (1000 IU/kg) was administered i.v. for anticoagulation. Each heart was quickly removed, washed in ice-cold Krebs–Henseleit solution (sodium chloride 115 mmol/L, potassium chloride 5.0 mmol/L, magnesium sulfate 1.2 mmol/L, sodium hydrogen carbonate 25 mmol/L, monobasic potassium phosphate 1.2 mmol/L, calcium chloride 1.5 mmol/L, and glucose 11 mmol/L) and then placed in a nonrecirculating Langendorff apparatus for perfusion with Krebs–Henseleit solution at a constant pressure of 80 mmHg at 37°C oxygenated by 95% oxygen and 5% carbon dioxide. Mounting of hearts in the Langendorff apparatus: The hearts were rapidly transferred to the Langendorff apparatus. The glass perfusion cannula was carefully inserted into the dorsal aorta in the retrograde direction, avoiding passage into the pulmonary artery via the ductus arteriosus. The aorta was then secured to the glass cannula using cotton ligatures tied proximally to the origin of the right brachiocephalic trunk. The positioning of the tip of the cannula distal to the aortic valves was confirmed by palpation. This ensured that all the perfusate passing through the glass perfusion cannula was directed through the coronary circulation.

Each heart underwent an initial 10 min of baseline normal perfusion and was subjected to perfusion at 37°C for 45 min. The hearts were then randomly divided into three groups: Control group (normal calcium perfusion) (1.5 mmol/L), high calcium group (high calcium perfusion) (3.3 mmol/L) and verapamil group (high calcium plus verapamil perfusion) (3.3 mmol/L calcium +3 µmol/L verapamil). For measurement of arrhythmias, the ECG was continuously monitored during the entire perfusion period and the incidence of arrhythmias was evaluated.

### Measurement of ECG and Determination of Arrhythmia Score

Anti-arrhythmic properties of verapamil were determined in an animal model of ischemia-induced arrhythmia or in the presence of Bay K8644 or heptanol or Gap 26, respectively. The occurrence of cardiac arrhythmias throughout the 45 min was compared by ECG recording. For analysis of arrhythmia in Langendorff-perfused rat heart, each rat heart was continuously monitored with a positive electrode attached to the heart and a negative electrode to the aorta. After 10 min of a baseline normal perfusion period, incidences of arrhythmias under different concentrations of Ca^2+^ in the initial 45 min of perfusion period were compared. To enable a good quantitative comparison, 45 min of an ischemia period were divided into 15 3-min intervals in an *in vivo* arrhythmia evaluation and 45 min of a perfusion period was divided into 15 3-min intervals in an *ex vivo* arrhythmia investigation. Arrhythmia scores were evaluated as described previously [10]. PVC ≤10/3-min period was recorded as 0; 10–50 PVC/3-min period was recorded as 1; ≥50 PVC/3-min period was recorded as 2; 1 episode of VF/3-min period was recorded as 3; 2–5 episodes of VF/3-min period was recorded as 4; and ≥5 episodes of VF/3-min period was recorded as 5.

### Examination of Cx43 Immunostaining

Before immunohistochemical examination, 3-µm slices from pretreated myocardium tissue were placed in a bathing solution of 3% H_2_O_2_ and 60% methanol phosphate-buffered saline (PBS) for 30 min and then treated with 0.01 mol/L sodium citrate buffer at 95°C in a microwave oven for 13 min (antigen retrieval). Thereafter, specimens were treated with 5% normal goat serum and 5% bovine serum albumin in PBS. Before each step, sections were rinsed three times in PBS buffer, incubated with primary antibodies (Anti-Connexin43 Antibody, mouse antirat, ab11369). After rinsing with PBS, sections were incubated with the corresponding secondary biotinylated antibodies (Histostain- plus Kit; ZSGB-BIO Company, China) for 1 hr at room temperature. A streptavidin/horseradish peroxidase complex was then applied as a detection system (1∶100 dilution) for 1 hr. Finally, staining was developed with 3,3′-diaminobenzidine tetra-hydrochloride (DAB kit; ZSGB-BIO Company, China) in 0.05 mol/L Tris-HCl buffer and 0.1% H_2_O_2_. Negative control sections were incubated without the primary antibody. All dates in this study were analyzed by software “Image Pro Plus” (Media Cybernetics Corporation, Washington, DC).

### Determination of Cx43 Protein Expression by Western Blotting

Heart tissues were lysed in buffer (1 mM each: antipain, benzamidine, leupeptin, pepstatin A, and phenylmethyl sulfonylfluoride (PMSF), 1% sodium dodecyl sulfate (SDS), and 5 mM ethylenediaminetetraacetic acid (EDTA)). Equal amounts of protein (40 µg protein/lane) were electrophoresed on a 12% SDS-polyacrylamide gel and transferred to nitrocellulose membranes (Pall Corporation, Ann Arbor, MI, USA). Membranes were blocked at room temperature with BSA (3% wt/vol) in Tris-buffered saline containing Tween 20 (0.5% vol/vol; TBS-T) for 1 h and then incubated with an antibody against Cx43 (1∶3000), tubulin (1∶3000) overnight at 4°C. Horseradish peroxidase-conjugated secondary antibodies were incubated at room temperature for 1 h and immune complexes were visualized by an enhanced chemiluminescence (ECL) Western Blotting Detection System (Bio-Rad, Hercules, CA, USA) and quantified using Quantity One Software (Bio-Rad).

### Statistical Analysis

Data were presented as mean ± SEM of *n* independent experiments and were subjected to one-way ANOVA followed by Tukey post hoc test. Incidence of arrhythmia was compared using χ^2^; *P*<0.05 was accepted as statistically significant.

## Results

### Effect of Verapamil on Ischemia-induced Arrhythmias

**Figure 1:**
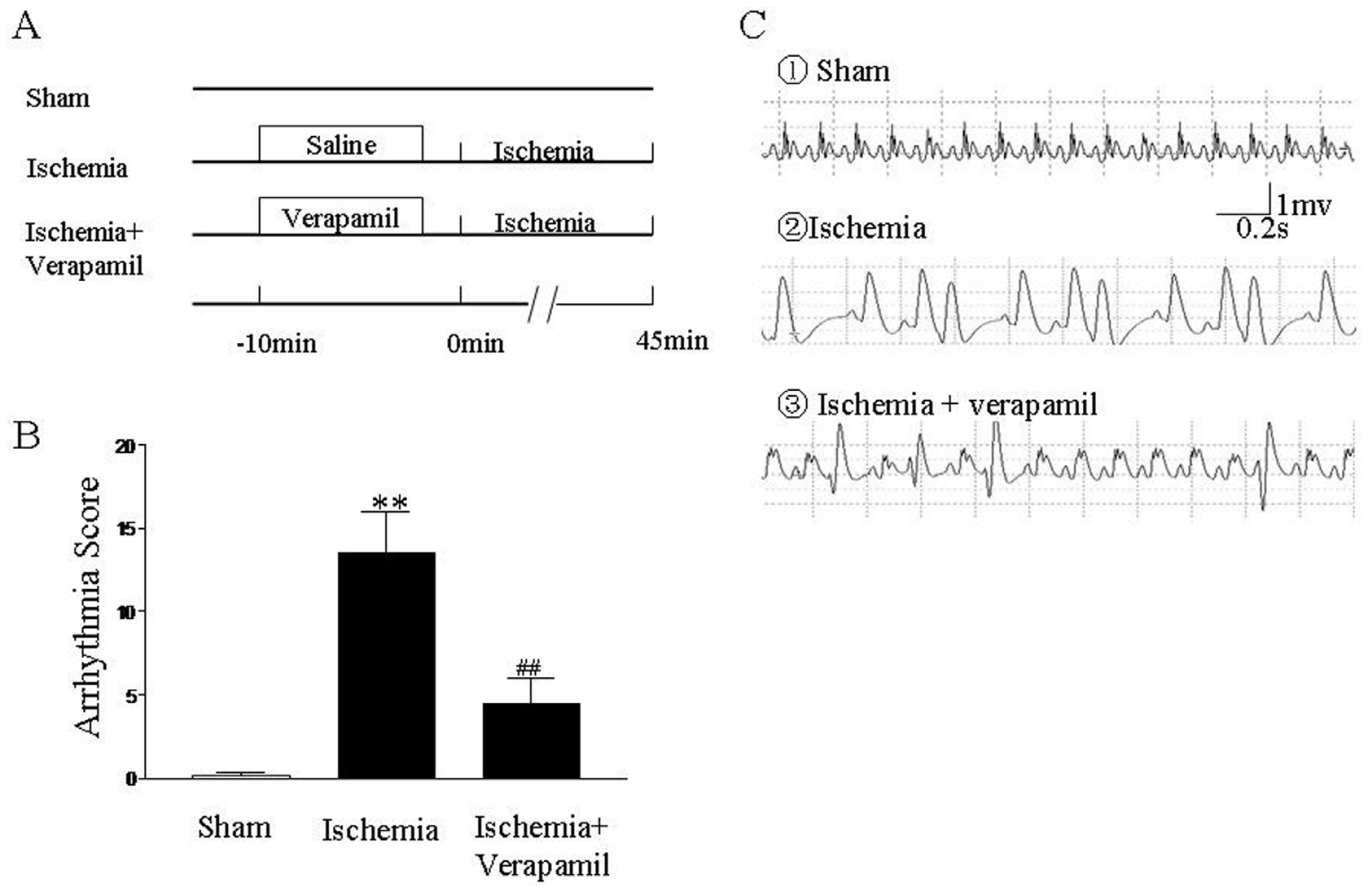
Anti-arrhythmic effect of verapamil in an animal model of ischemic arrhythmia. (A) Protocols for arrhythmia study. (B) Total arrhythmia scores during 45 min of ischemia period. (C)Representative tracings of ECG recordings. Verapamil, a calcium channel antagonist, at 1 mg/kg was i.v. injected into a femoral vein 10 min prior to ischemia. Anti-arrhythmic property of verapamil was evaluated. **P<0.01 vs. sham group, ^##^ *P*<0.01 vs. ischemia group. All values are expressed as mean ± SD, *n = *10 in each group.

In order to ascertain the anti-arrhythmic property of verapamil, SD rats were subjected to 45 min of ischemia. Verapamil (1 mg/kg) significantly decreased the incidence of ventricular arrhythmias including PVC, VT and VF for 45-min coronary artery occlusion (Table 1). As shown in Figure 1, total arrhythmia scores were significantly increased when the heart was subjected to ischemia (*P*<0.01 vs. sham). Verapamil (1 mg/kg) significantly prevented the enhancement of total arrhythmia scores induced by ischemia (*P*<0.01 vs. ischemia). Results indicate that verapamil exerts an anti-arrhythmic property.

**Table 1:** Effect of verapamil on incidence of ventricular arrhythmias during 45 min ischemia.

x	n	VPC n (%)	VT n (%)	VF n (%)
Control(vehicle)	10	9 (90)	8 (80)	5 (50)
Verapamil(1 mg/kg)	10	7 (70)*	5 (50)**	1 (10)**

Note. n = number of animals; control (vehicle) is saline solution. VPC, ventricular premature contraction; VT, ventricular tachycardia; VF, ventricular fibrillation. *P<0.05, **P<0.01 vs. the control group.

### Effect of Verapamil on Bay K8644-induced Arrhythmias

**Figure 2:**
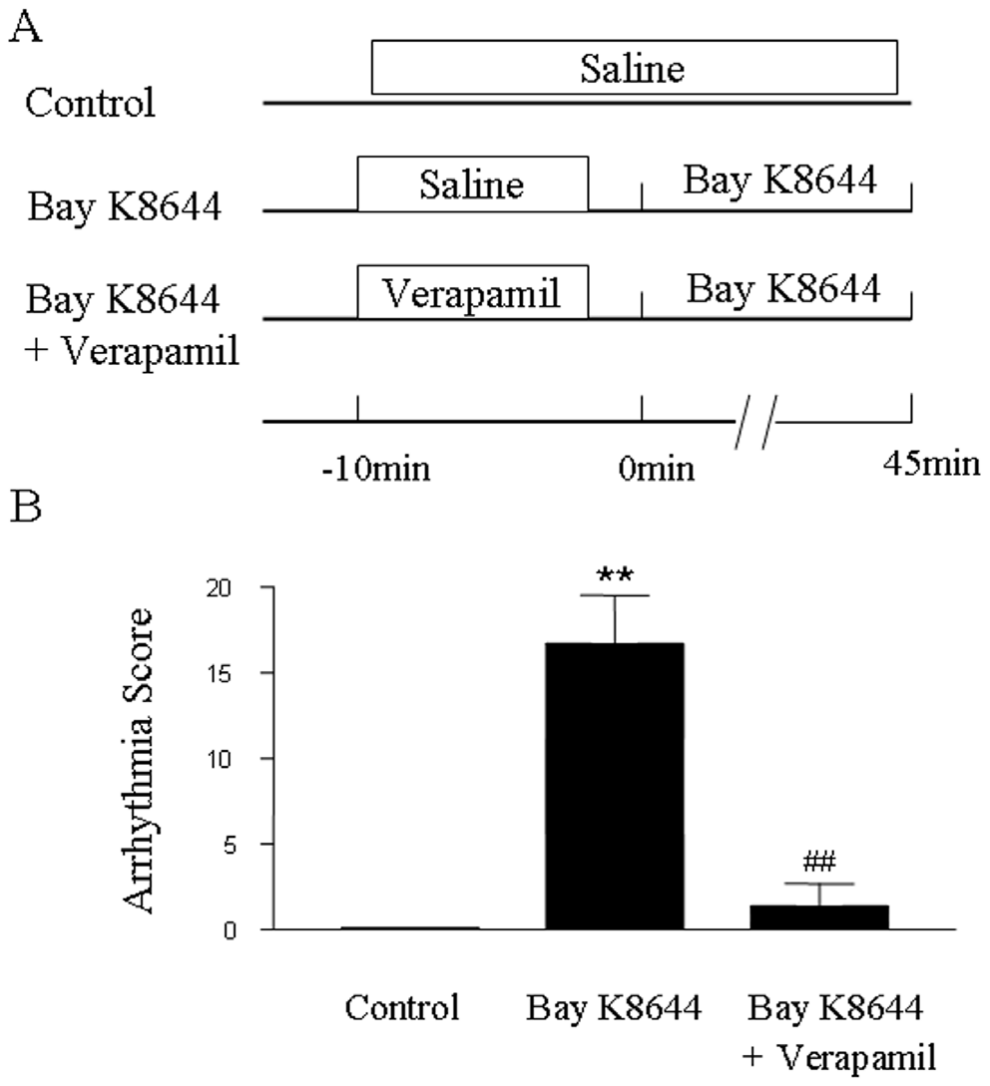
Effects of verapamil on arrhythmias induced by Bay K8644. (A) Protocols for arrhythmia study. (B) Total arrhythmia scores during 45 min of Bay K8644 (L-type calcium channel agonist, 0.1 mg/kg) administration. Verapamil at 1 mg/kg was injected i.v. into a femoral vein 10 min prior to Bay K8644. Anti-arrhythmic property of verapamil was evaluated in the presence of Bay K8644. **P<0.01 vs. control, ^##^ *P*<0.01 vs. Bay K8644. All values are expressed as mean ± SD, *n = *12 in each group.

In order to further ascertain the anti-arrhythmic property of verapamil, the effects of verapamil on Bay K8644-induced arrhythmias were determined. Bay K8644, an L-type calcium channel agonist, significantly increased arrhythmia score at 0.1 mg/kg as shown in Figure 2. Verapamil (1 mg/kg) significantly prevented the enhancement of total arrhythmia scores induced by Bay K8644 (*P*<0.01). The results indicate that verapamil exerts an anti-arrhythmic property via inhibition of L-type calcium channel.

### Effect of Verapamil on Hemodynamics in Rats Subjected to Myocardial Ischemia

Hemodynamic parameters were continuously recorded. Table 2 summarizes HR, MABP and LVSP in all groups determined at baseline, 1 minute after ischemia, and 45 minutes after ischemia. There were no significant differences in HR during ischemia, but MABP and LVSP significantly decreased during ischemia. Administration of verapamil (1 mg/kg) prior to myocardial ischemia further significantly reduced HR, MABP and LVSP.

**Table 2:** Effect of verapamil on hemodynamics in rats subjected to myocardial ischemia.

Parameters	Groups	Before ischemia	1 min after ischemia	45 min after ischemia
HR (beats/min)	Sham	387±12	390±16	385±15
	Ischemia	386±19	391±16	389±15
	Verapamil+Ischemia	384±14	367±19*	356±22**
MABP (mmHg)	Sham	92.2±7.3	90.4±8.7	90.1±6.9
	Ischemia	92.6±7.5	81.8±5.9*	67.2±8.5**
	Verapamil+Ischemia	91.8±6.8	69.9±6.7** ##	50.7±6.9** ##
LVSP (mmHg)	Sham	132.4±5.2	131.8±8.7	130.1±6.3
	Ischemia	131.9±9.4	92.7±7.8**	80.6±7.7**
	Verapamil+Ischemia	134.6±8.6	80.4±8.7** #	70.2±8.7** ##

n = 6 in each group,

* *P*<0.05,

** *P*<0.01 vs. ischemia before.

# *P*<0.05,

## *P*<0.01 vs. ischemia group. HR, heart rate; MAP, mean arterial blood pressure; LVSP, left ventricular systolic pressure.

### Effect of Verapamil on High Calcium-induced Arrhythmias

**Figure 3:**
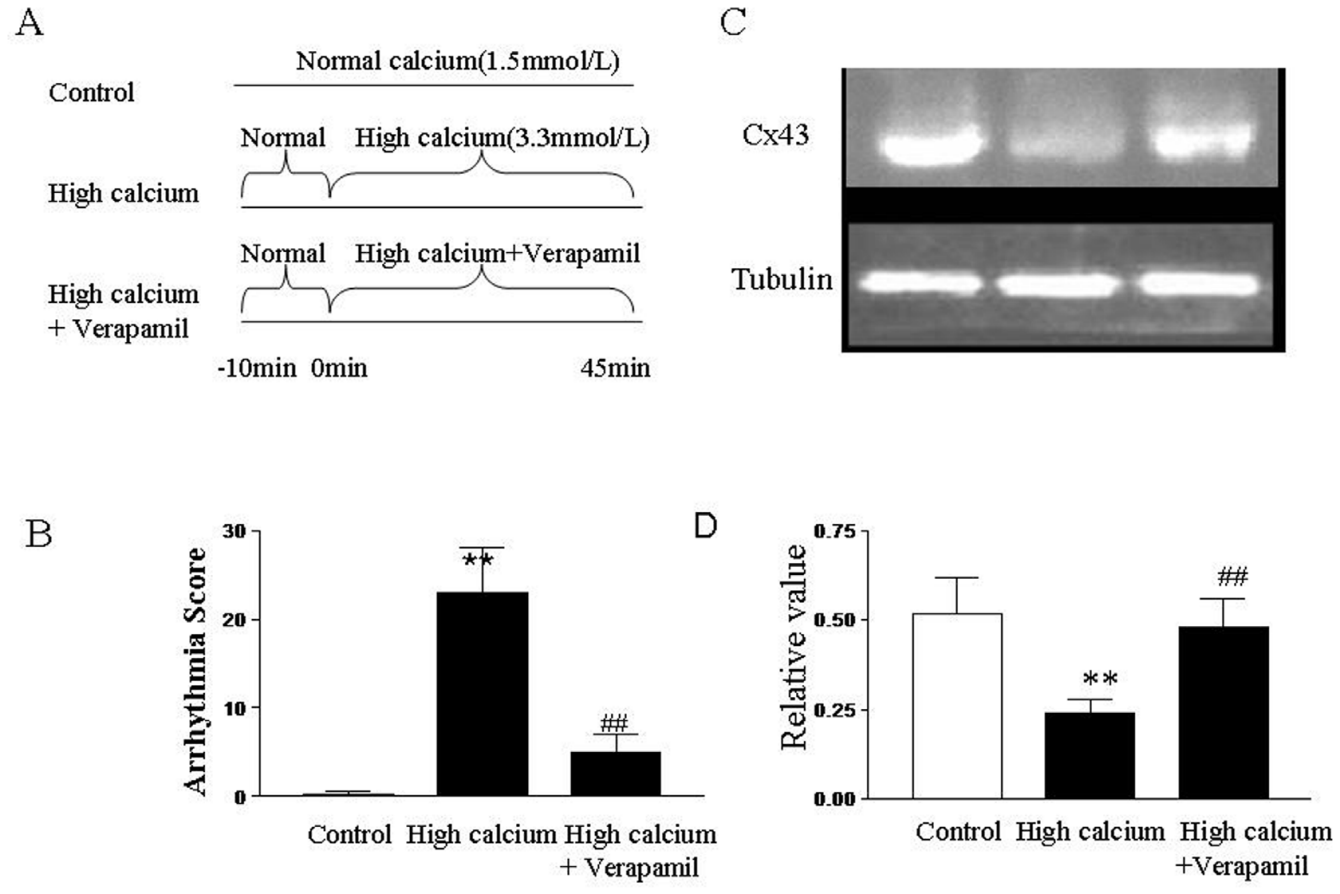
Effects of verapamil on cardiac arrhythmias and expression of Cx43 protein in isolated rat hearts subjected to perfusion with high concentration of calcium. (A) Protocols for *ex vivo* arrhythmia study. (B) Total arrhythmia scores during 45 min of perfusion period. Anti-arrhythmic property of verapamil at 0.3 µmol/L was evaluated. (C) Immunoblots of Cx43 expression. Representative immunoblots of samples from rat ventricles subjected to 45 min of perfusion with high concentration of calcium in the absence and presence of verapamil. (D) Quantitative densitometric analysis of Cx43 protein with tubulin as an internal standard. All values are expressed as mean ± SD. *n* = 12 for arrhythmia analysis. *n* = 8 for Western blot analysis. Control: normal calcium (1.5 mmol/L) perfusion group; high calcium: high calcium (3.3 mmol/L) perfusion group; high calcium+verapamil: high calcium (3.3 mmol/L) perfusion in the presence of verapamil (0.3 µmol/L) group. **P<0.01**vs. control, ^##^ *P*<0.01 vs. high calcium group.

To further determine the mechanism underlying the effect of verapamil, ECG recordings and immunoblot analysis were performed in isolated perfused hearts. As shown in Figure 3B, high calcium perfusion (3.3 mmol/L) significantly increased the total arrhythmia scores of the isolated rat heart (*P*<0.01 vs. control). As demonstrated in Figures 3C and 3D, high calcium conditions (3.3 mmol/L) also remarkably attenuated expression of the Cx43 protein compared with the normal calcium perfusion group (1.5 mmol/L) (*P*<0.01). Interestingly, verapamil not only significantly decreased high calcium-induced cardiac arrhythmias (Figure 3B) but also prevented the reduction in Cx43 protein expression (Figures 3C and 3D). The results suggest that cardiac arrhythmias induced by high calcium perfusion may be attributed to Cx43 destruction induced by calcium overload and anti-arrhythmic effect of verapamil is accompanied by preservation of Cx43 protein.

### Effect of Verapamil on Cx43 Immunostaining Following Myocardial Ischemia

**Figure 4:**
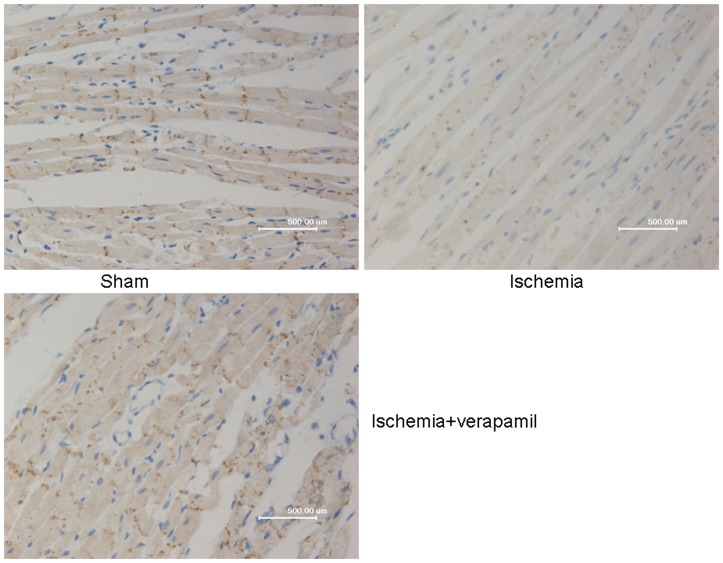
Effects of verapamil on Cx43 immunostaining in an animal model of ischemia. In rat hearts subjected to 45 mins ischemia, most Cx43 signals disappeared and the location of vestigial Cx43 was distributed sparsely at the intercalated disks and most of the Cx43 signals were distributed on the flank of myocardial cells. In contrast, verapamil (at 1 mg/kg was i.v. injected into a femoral vein 10 min prior to ischemia) resulted in a marked preservation of Cx43 signals in gap junctions.

As shown in Figure 4, localization of immunoreactive signals in the sham group was restricted to intercellular junctions where gap junctions and intercalated disks were located. However, in rat hearts subjected to 45 min ischemia, most Cx43 signals disappeared and the location of vestigial Cx43 was distributed sparsely at the intercalated disks and most of the Cx43 signals were distributed on the flank of myocardial cells. Both the mean intensity and area of Cx43 were significantly decreased in the ischemia group. In contrast, verapamil (1 mg/kg) resulted in a marked preservation of Cx43 signals in gap junctions. The amount of Cx43 signals at intercellular junctions was approximately four times higher in ischemic rat hearts that were given verapamil compared with the ischemia group in which rats had undergone 45 min of ischemia. These results suggest that verapamil played an important role in the preservation of Cx43 when rat hearts were subjected to 45 min ischemia in vivo.

### Effect of Verapamil on Expression of Cx43 Protein Following Myocardial Ischemia

**Figure 5:**
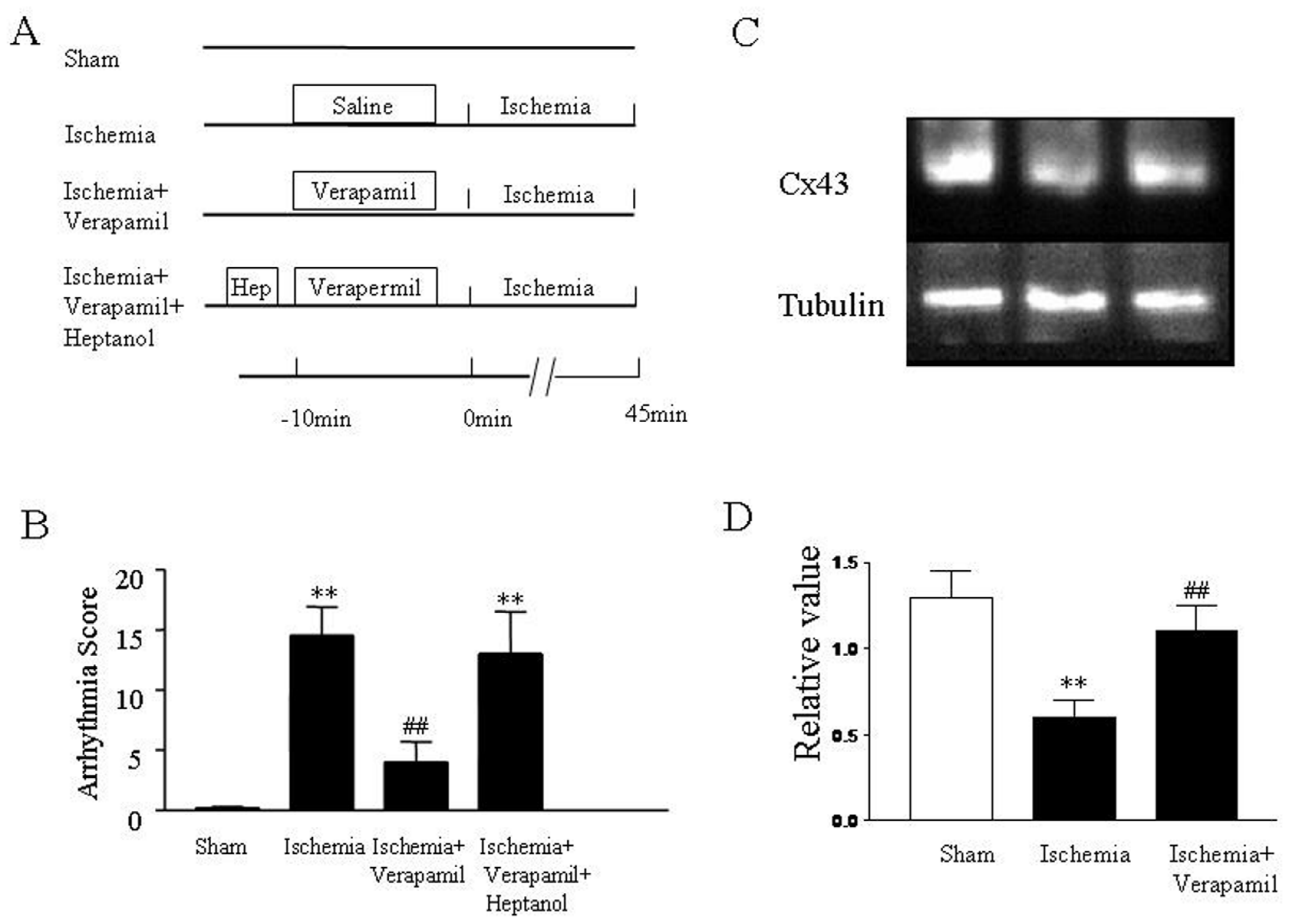
Effects of verapamil on cardiac arrhythmias and expression of Cx43 protein in an animal model of ischemia. (A) Protocols for arrhythmia study. Anti-arrhythmic property of verapamil was evaluated following myocardial ischemia in the absence and presence of heptanol or Gap 26. Verapamil (1 mg/kg) was injected i.v. into a femoral vein 10 min prior to ischemia. Heptanol (a Cx43 uncoupler, 0.1 mg/kg) or Gap 26(a Cx43 inhibitor, 0.1 mg/kg) was injected i.v. into a femoral vein 5 min prior to verapamil. (B) Group results showing the total arrhythmia scores in different treatment groups. **P<0.01 vs. sham group, ^##^ *P*<0.01 vs. ischemia group, and ^‡^ *P*<0.01 vs. ischemia +verapamil group. All values are expressed as mean ± SD, *n = *12 in each group. (C) Immunoblots of Cx43 expression. Representative immunoblots of samples from rat heart subjected to 45 min of ischemia in the absence and presence of verapamil. (D) Quantitative densitometric analysis of Cx43 protein with tubulin as an internal standard. All values are expressed as mean ± SD; *n* = 8 for Western blot analysis. **P<0.01**vs. sham group, ^##^ *P*<0.01 vs. ischemia group.

To further determine the relationship between the anti-arrhythmic property of verapamil and Cx43, ECG recordings and immunoblot analysis were performed in the heart subjected to myocardial ischemia. Results demonstrated that the anti-arrhythmic property of verapamil was almost totally abolished by prior administration of heptanol, a Cx43 uncoupler, and Gap 26, a Cx43 channels inhibitor (Figure 5A and B). Meanwhile, myocardial ischemia remarkably attenuated the expression of the Cx43 protein compared with sham group (*P*<0.01) and was reversed significantly by verapamil (Figures 5C and 5D). These results suggested that Cx43 protein underwent reduction when the heart was subjected to ischemia, and verapamil preserved Cx43 protein against ischemia. Together with the previously mentioned results, this study indicates that verapamil may exert its anti-arrhythmic property via preservation of the Cx43 protein against ischemia insult.

## Discussion

In the present study, administration of verapamil prior to ischemia revealed a significant anti-arrhythmic property, and this effect may be related to the preservation of Cx43 protein via inhibition of L-type calcium channel and oxygen consumption. This is based on the following observations: *1)* myocardial ischemia and high concentration of extracellular Ca^2+^ may trigger intracellular calcium overload, thus leading to decrease of Cx43 protein, which favors the occurrence of cardiac arrhythmias and *2)* anti-arrhythmic effects of verapamil were abolished by heptanol, a Cx43 uncoupler, or Gap 26, a Cx43 channels inhibitor, and accompanied by protection of Cx43 protein against ischemic insults via inhibiting L-type calcium channels. *3*) verapamil reduced heart rate, ventricular contractility, and blood pressure, it should exhibit anti-arrhythmic effects through inhibition of oxygen consumption.

In the present investigation, direct evidence suggests that verapamil significantly attenuates cardiac arrhythmias induced by myocardial ischemia, Bay K8644, and high calcium perfusion via inhibition of calcium channel. Numerous studies have demonstrated that myocardial ischemia is associated with a marked accumulation of Ca^2+^, calcium overload and oscillation of calcium, which favors the occurrence of arrhythmias [13], [14]. A previous study also demonstrated that abnormal elevation of Ca^2+^ and/or calcium overload as well as its subsequent alterations may account for hypokalemia-induced arrhythmias [15]. Thus, strategies focusing on attenuation of calcium overload may result in anti-arrhythmic effects [16], [17]. The clinical use of calcium channel blockers as class IV anti-arrhythmic drugs supports this concept.

Cx43 is functionally associated with calcium and its open/closed conformations can be regulated by calcium [18]. Research by DeMello demonstrated that high Ca^2+^ inhibits the function of gap junctional channels and abolishes cell-to-cell communications [19]. Another study demonstrated that abnormal expression and function of gap junctions may be modulated by calcium overload [20], indicating that there is a close link between gap junction and intracellular Ca^2+^. In this regard, a previous study demonstrated a significant decrease in phosphorylation status of Cx43 in calcium-overloaded ventricular muscles [21]. These studies support the concept that functional phosphorylated isoforms of Cx43 (P-Cx43) are downregulated by calcium overload. In our present study we provided direct evidence that high Ca^2+^ perfused rat heart was more susceptible to cardiac arrhythmias and was accompanied at the same time by loss of Cx43 protein. This indicates that intracellular calcium overload-induced attenuation of Cx43 protein may account for the initiation of high calcium-induced arrhythmias. This concept is also supported by a previous study in which hypokalemia-induced arrhythmias were attributed to hypokalemia-induced high calcium and/or calcium overload accompanied by a decreased number of gap junctions according to immunochemistry [15].

In our present study we investigated the anti-arrhythmic effect of verapamil during myocardial ischemia. Our study demonstrated for the first time that the anti-arrhythmic effect of verapamil was almost totally abolished by prior administration of heptanol or Gap 26, suggesting that verapamil may exert its anti-arrhythmic property via Cx43 protein.

It has been reported that Gap26 rapidly inhibits connexin hemichannels (2.6 min) and only with some latency blocks gap junctions (30–40 min), this diverse response pattern is intriguing. With regard to hemichannels, the rapid response time suggests a direct action of Gap26 on freely accessible sites of Cx43 located in the extracellular loop. As to gap junction channels, the slow on-response suggests a tortuous diffusion pathway for Gap26 to reach the sites of action, i.e. extracellular loop, the narrow intercellular clefts between adjacent cells acting as hindering barriers [22]. So it seems to be reasonable to doubt whether the gap junction coupling was sufficiently suppressed and the effect was not attributed to inhibition of gap junction channels. In the present study, we have two explanations for arguing against this doubt. Firstly, in the present in vivo study, Gap26 was given 15 min before the onset of ischemia (ischemia for 45 min), that is to say the action time of Gap26 is nearly 60 min, this action time is largely more than 30–40 min latency, which was obtained from an in vitro study with cell pairs by Desplanteza T. et al [22], and in this study, they also documented that the efficacy and speed of inhibition of Gap26 is likely more quickly in vivo than in vitro. Secondly, Gap26 at a very low dose (1 µg/kg) was used to effectively reduce infarct size in the previous study [23], while in the present study, Gap26 at a relatively larger dose (100 µg/kg) was used, which may have a larger diffusion ability to carry out the block of gap junction channels.

To testify that verapamil can preserve Cx43 protein, the immunohistochemical analysis was employed. It was found that ischemia-induced redistribution and reduced immunostaining of Cx43 protein were prevented by verapamil. The result suggests that verapamil may play an important role in the preservation of Cx43 when rat hearts were subjected to 45-min ischemia in vivo. To further testify that verapamil can preserve Cx43 protein during high calcium perfusion or during myocardial ischemia, immunoblot analysis was performed in the heart subjected to high calcium perfusion or myocardial ischemia. We found that both high calcium perfusion and myocardial ischemia remarkably attenuated the expression of the Cx43 protein and the latter was reversed significantly by verapamil. These results further suggest that verapamil preserved Cx43 protein against high calcium and myocardial ischemia. Together with the above-mentioned results, this study indicates that verapamil may exert its anti-arrhythmic property by preserving Cx43 protein against myocardial ischemia insult.

In the present study, one of the limitations is that only the total ventricular Cx43 protein was determined, it is known that the total Cx43 does not necessarily represent the functional status of Cx43 channels. Ischemic insults are accompanied not only with reduced Cx43 expression, but also redistribution and altered phosphorylation status. For this reason, it is utmost important to further elaborate on the phosphorylation status of Cx43 and the ischemia-induced Cx43 remodeling with and without verapamil administration. Our future study is needed to clarify these specific questions.

It was recently reported that bepridil, another class IV anti-arrhythmic drug, increases intercellular coupling, and the effects of bepridil on conduction were well established. These data suggest that the effect of class IV anti-arrhythmic drugs is beneficial to the stabilization of conduction [24]. Decades ago, verapamil was shown to have anti-arrhythmic properties against ischemia infarction-induced ventricular tachycardia [25]. Initially, this was related to intracellular calcium handling because verapamil was known to block the L-type calcium channel. Mechanistically, verapamil was responsible for preventing the (local) initiating beats of the arrhythmia. Continuation of the arrhythmia was thought to be based on the slowing of conduction, conduction block and reentry. An action of verapamil that prevented slowing of conduction was also suggested but never proven. In this study, the two lines of action start being connected. We have shown a possible relation between blocking calcium handling by verapamil and preservation of the conduction by maintaining Cx43 levels. As for the conduction parameters that have not been mentioned, further studies are needed to verify this relation.

In summary, our study provides the initial evidence that verapamil may confer its anti-arrhythmic effects via modulating the Ca^2+^-Cx43 pathway. This will shed light on a new mechanism for the anti-arrhythmic property of calcium channel blockage. Data from this investigation suggest that the Ca^2+^-Cx43 pathway may be regarded as a potentially important therapeutic target against ischemic arrhythmias.
